# Macrophage immunomodulatory activity of *Acanthopanax senticousus polysaccharide* nanoemulsion via activation of P65/JNK/ikkαsignaling pathway and regulation of Th1/Th2 Cytokines

**DOI:** 10.7717/peerj.12575

**Published:** 2021-12-24

**Authors:** Xianghui Li, Zhiqiang Zhang, Zhenhuan Guo, Li Zhao, Yonglu Liu, Xia Ma, Qigai He

**Affiliations:** 1 State Key Laboratory of Agricultural Microbiology/College of Veterinary Medicine, Huazhong Agricultural University, Wuhan, China; 2 Medicinal Engineering Department of Henan University of Animal Husbandry and Economy, Zhengzhou, China; 3 Jiangsu Key Laboratory for Pharmacology and Safety Evaluation of Chinese Materia Medica, Nanjing University of Chinese Medicine, Nanjing, China; 4 Research Center for the inheritance and innovation of Chinese veterinary medicine classic prescriptions, Henan University of Animal Husbandry and Economy, Zhengzhou, China

**Keywords:** Nanoemulsion, *Acanthopanax senticosus polysaccharide*, Immune responses, T lymphocyte, Pseudo-ternary phase diagram

## Abstract

Nanoemulsions (NE) are used widely in pharmaceutical drug formulations and vaccine preparation, and *Acanthopanax senticousus polysaccharide* (ASPS) is a natural bioactive compound with immunostimulatory activity. Therefore, NE-loaded ASPS is expected to provide immunological enhancement for effective treatment. In the present study, *Acanthopanax senticousus polysaccharide* (ASPS was encapsulated into nanoemulsions, the resultant ASPS–NE were coated with a negative charge, and the immune enhancement mechanism of these ASPS-NE formulations was analyzed. The immunosuppressive animal models (70 ICR mice, male) for the study were established using cyclophosphamide. In addition, the activation of splenocyte proliferation, phagocytosis of the macrophages, the ratio of CD4^+^ to CD8^+^, the concentrations of the cytokines in serum, Western blot analysis was used for the analysis of the P65/JNK/ikk α signaling pathway in the peritoneal macrophage s. The results revealed that the ASPS-NE could stimulated the proliferation of splenocytes and enhance immunity. The ASPS-NE induced the expression of different cytokines (TNF-α, IFN-γ, IL-2, and IL-6), could activate the expressions of P65, JNK, and ikkα, and regulated the Th1/Th2 cytokines. These findings demonstrated the potential of ASPS-NE formulations for drug delivery and to induce potent and sustained immune responses.

## Introduction

*Acanthopanax senticosus* (AS) is a herbal medicine that is used extensively for the treatment of anti-inflammatory, antioxidative, antifatigue, and hypoglycaemic effects (Aja et al., 2020). *Acanthopanax senticousus polysaccharide* (ASPS) is a major active ingredient extracted from AS, with several biological and pharmacological activities, such as anti-inflammatory activity and immunoregulation (Han et al., 2014; Han et al., 2003). ASPS is reported as a natural source of immune polysaccharides that exhibit favorable bioactivities such as low toxicity and immunostimulating activity. The components of ASPS include rhamnose, xylose, glucuronic acid, fructose, and glucose, among others (Zhu et al., 2013). ASPS has been used as an immunological adjuvant in veterinary clinical studies. ASPS promotes non-specific cell-mediated immunity in the body along with improving the antiviral capacity, which renders it suitable for application in the relevant fields. However, the clinical application of ASPS has several limitations, such as the local and systemic side-effects when used in large dosage, rapid metabolism, poor pharmacological or biological function, and poor efficiency against certain antigens (Li, Lee & JJCSr, 2018; Xiao et al., 2018). In addition, certain alkali-soluble polysaccharides of ASPS are difficult to absorb. Therefore, searching for an appropriate dosage formulation for ASPS exhibiting improved biological activity, long-term release, and enhanced immune effect at reduced doses is of particular importance.

Nanoemulsion (NE) are formed through self-assembly when mixed with the oil/aqueous phase, co-surfactant, or surfactant, and represent the colloidal nanocarrier systems exhibiting thermodynamic stability and the ability to resolve hydrophobic bioactive compounds  (Haider et al., 2020; Leão et al., 2019). Currently, NE represent a novel class of drug carriers, offering numerous advantages, such as simple synthesis process, low cost, and high strength and stability (Mehmood et al., 2018; Thompson et al., 2018). In addition, NE increase the solubility of fat-soluble drugs, a property that has been applied widely in the production of various chemical and biological materials (Fratter, Biagi & Cicero, 2019; Hosny et al., 2019).

In the present work, the role of ASPS-NE in the activation of macrophages and lymphocyte uptake was demonstrated in animal models. The immunosuppressive animal models were established using cyclophosphamide. The activation of splenocyte proliferation, phagocytosis of the macrophages, the ratio of CD4^+^ to CD8^+^, the concentrations of cytokines in serum and protein expression related to the P65/JNK/ikk α signaling pathway were analyzed at selected time points.

## Materials and Methods

### Preparation of ASPS-NE

#### Preparation of ASPS loaded k-Carrageenan beads(k-Car-ASPS)

k-Car-ASPS was prepared as follows, 0.1 g of k-Car was added into 50 ml of distilled water and the temperature was adjusted to 80 °C, the prepared contents were stirred using magnetic stirrer until k-Car was dissolved completely. A stock solution of curcumin was prepared in ethanol at a concentration of 1 mg/ml. In parallel 50 mg of ASPS was dissolved in 5 ml of aqueous solution and added to the above stirred solution. The solution was again re-stirred for 1 h at 80 °C to obtain a clear, viscous and homogeneous solution without bubble. Finally, the bead production process was carried out using the interphase technique. The above-prepared mixture containing Carrageenan and curcumin were dropped through a syringe into the beaker containing sunflower oil in aqueous phase (5% KCl). Beads were passed through the oil layer and it was collected in the aqueous phase;resulting in spherical beads left behind in the aqueous phase for an hour.

#### Preparation of ASPS-NE

The ASPS-NE formulations were synthesized using the method of pseudoternary phase diagram (Yu et al., 2020; Zhao et al., 2020). On the basis of the nanoemulsion regions in the phase diagram, ethanol was selected as the co-surfactant (Germany BASF Co., Ltd). Tween-80 and span-80 (Damao Chemical reagent factory, Shanghai, China) were selected as surfactants (Cao et al., 2019; Wang et al., 2018). Liquid paraffin (Damao Chemical reagent factory, Tianjin, China), Isopropyl myristate (IPM, Lanxi Wumart Chemical Co., Ltd., Zhejiang, China) was selected as the oil phase, *Acanthopanax senticosus polysaccharide* (ASPS: purity =98%, average MW =30.51 kDa) was provided by Shanghai Yuanye Biotechnology Co., Ltd. First, k-Car-ASPS was dissolved in deionized water, and the resulting aqueous solution was then mixed with ethanol and liquid paraffin, followed by the addition of respective amounts of Tween-80, Span 80, and IPM. The clear and transparent nanoemulsion was obtained after magnetic stirring. The final NE formulation comprised 46.2% Tween-80, 15.4% Span 80, 7.6% ethanol, 15.4% liquid paraffin, 11.5% IPM, and 3.9% aqueous ASPS solution.

### Characterization of the prepared ASPS-NE

#### Identification of the appearance and type

The appearance of the prepared ASPS-NE was identified in terms of homogeneity, transparency, and clarity. In addition, the ASPS-NE type was identified by adopting Methylene blue and Sudan red spreading speed methods (Solarbio, Beijing, China).

#### Identification of morphology and particle size

Transmission electron microscopy (Tecnai G220, USA) was employed to observe the shape of the prepared ASPS-NE. Prior to characterization, the ASPS-NE formulations were diluted in IPM to a final volume of 500 µL. The diluted nanoemulsion droplets were dried with filter paper, and then dropped 20 mg /ml phosphotungstic acid solution (pH = 7.4) and negatively stained on the copper net for 3 min. The nanoemulsion droplets were dried naturally. the morphology of nanoemulsion droplets was observed under TEM .This experiment was repeated in six times. The size distribution, polydispersity index (PDI), and zeta potential of the freshly prepared ASPS-NE formulations were determined using the NE particle size analyzer (Zetasizer 3000 H (Malvern Instruments Ltd, Worcestershire, UK).

#### *In vitro* release determination

The exact same amounts (calculated according to the content of ASPS) of ASPS-NE and ASPS were used. The dialysis tube was placed inside a hanging basket, which was placed in a dissolution cup containing 350 ml of PBS. The saline solution was maintained at (37 ± 0.5) °C and 100 r/min, and 3.5 ml samples were retrieved from the cup at regular intervals, along with the simultaneous supplementation of the released medium. The retrieved sample was diluted in PBS to a final volume of 7 ml, and the suspension was subjected to 20 min of centrifugation at 10,000 rpm and 4 °C. An ultraviolet spectrophotometer was employed to measure the absorbance (OD) value, followed by the calculation of the cumulative release percentage.

#### Stability

The cold–hot cycle experiment (4 °C, 60 °C), the lighted test (4500 ± 500 lx), and the accelerated stability test (40 ± 2 °C, 75% ± 5% RH) were conducted to evaluate the stability of the prepared ASPS–NE formulations.

### The immunological enhancement effect of ASPS-NE formulations in immunosuppressive mice

#### Animals and immunizations

ICR mice (7-weeks-old, male, healthy,body weight (BW) 18∼22 g) were provided by the experimental Animal Center of Zhengzhou University. Each mice was subjected to one week of adaptive feeding prior to the initial immunization (license: SCXK (SU) 2020–0001),The mice were allowed to eat and drink freely, well ventilated, alternated between light and dark for 12h-12 h, and observed the health of the mice.

All animal experiments were conducted in accordance with the guidelines of the Animal Welfare and Ethics Committee (PZ2020003) and the care and use of laboratory animals (Henan University of Animal Husbandry and Economics IACUC; Approval ID: 2020BAD34B02). All mice were maintained climate controlled (temperature: 25 °C ± 1 °C; humidity: 40% ± 10%) and photoperiod controlled (12 h light–dark cycles) housing with free food and water.

Eighty mices were randomly divided into eight groups, and two mices per cage (*n* = 10). The specific grouping are as follows: blank group (NC), model group (MC, cyclophosphamide, 80 mg/kg/d), positive drug group (PC), ASPS-NE low-dose group (ASPS-NE(L), 31.25 mg/kg/d), middle dose group (ASPS-NE(M), 62.5 mg/kg/d), high-dose group (ASPS-NE(H), 125 mg/kg/d), NE group and ASPS group (62.5 mg/kg/d), respectively.

The specific administration methods are as follows: the pc group was injected with thymopentin (Beijing double-aigrettes; Pharmaceutical Co. Ltd.) at 10 mg/kg/d; ASPS-NE (H, M, L) groups were injected with ASPS-NE at 125, 62.5, 31.25 mg/kg/d , respectively; ASPS group was injected with ASPS at 62.5 mg/kg/d; the NE group was injected with NE at 0.2ml/d; the nc group was injected with normal saline at 0.2 ml/d. all the above groups were treated for 7 days. except for the nc group, all the other groups were injected with cyclophosphamide (CY, Beyotime Biotechnology Co., Ltd.) at a dose of 80 mg/kg/d on day 1,2.5, respectively.

The Mice was euthanized by pentobarbital (ip 100 mg/kg.w) after receiving the last dose and then put to death with cervical dislocation. Each animal was managed according to the guidelines of the local Ethics Committee and the Chinese law. Any anticipated or unexpected adverse events will be reported.

#### Splenocyte proliferation assay *in vivo*

Splenocyte activation was evaluated using the splenocyte proliferation assay as described in a previous study (Ren et al., 2017) .The animals were sacrificed 24 h after receiving the last dose to isolate splenocytes.

Freshly separated splenocytes (2.5 × 10^6^ cells/ml) from the seven groups were first cultured in RPMI-1640 solution (Sinopharm Chemical Reagent Co. Ltd., Shanghai, China), Concanavalin A (ConA, 10 µg /ml, Sigma Co.), or LPS (5 µg/ml) (Sigma Co.), and then inoculated into the wells of 96-well plates (100 µL/well) in triplicate. The plates were incubated at 37 °C in a 5% CO_2_ atmosphere for 44 h. cell proliferation was estimated using the CCK-8 method. The average OD value was determined at 450 nm using a microplate reader (Varioskan; Thermo Scientific). Each experiment was repeated four times.

#### Spleen and thymus index measurements

The animals were sacrificed 24 h after receiving the last dose to isolate the thymus and spleen, and then the organs were weighed. The spleen and thymus indices were calculated using the following equation:

Spleen index (mg/10 g) = spleen weight (mg)/BW (mg) ×10

Thymus index (mg/10 g) = thymus weight (mg)/BW (mg) ×10

#### Macrophages activation

Macrophage activation was detected using the neutral red uptake experiment. The cells obtained from each group were washed with Hanks’ solution and then subjected to 0.1% neutral red staining for 2 h. Subsequently, 5  × 10^4^/ml macrophage cell suspension was added to each well of the 96-well plates, and the plates were incubated at 37 °C in 5% CO_2_ conditions for 44 h. After the incubation, 100 µL of the neutral red solution was added to each well, followed by two washes with Hanks’ solution. Next, 100 µL of cell dissolution buffer (0.01% acetic acid and ethanol (1:1, v/v)) was added to each well for cell dissolution. Finally, the microplate (Varioskan; Thermo Fisher Scientific) was subjected to OD measurement at 540 nm. All the experiments were repeated four times.

#### ASPS-NE enhances the proliferation and differentiation of CD4^+^ and CD8^+^ T cells

The mice were sacrificed 24 h after receiving the last dose, and peripheral blood samples were collected in tubes containing the anticoagulant. In order to evaluate the proportions of CD3^+^CD4^+^/CD3^+^CD8^+^T cells, the cells were allowed to react with anti-CD3-APC, anti-CD4-PE, and anti-CD8a-FITC antibodies (BD, USA), followed by the analysis of the peripheral blood lymphocytes in a flow cytometer (Beckman CytoFLEX FCM).

#### Serum cytokine detection through ELISA

The serum levels of cytokines IFN-γ, TNF-α, IL-2, and IL-4 were determined using the corresponding ELISA kits (RD Co. Ltd.) in accordance with the manufacturer’s specifications. Each experiment was performed in triplicate.

#### Western Blot assay for P65/JNK/ikk α signaling pathway in peritoneal macrophages

The total protein in the peritoneal macrophages was extracted using the radioimmunoprecipitation assay (RIPA) lysis buffer (Boster Tech Co., Ltd, China) in accordance with the specific protocols recommended by the manufacturer. The protein contents were determined using the BCA method.

The proteins were separated by applying 40 mg of the protein lysate on an SDS-PAGE gel, and the separated proteins were transferred to 0.2-mm PVDF membranes. Subsequently, the membranes were blocked by incubation with Tris-buffered saline (TBS) supplemented with 5% bovine serum albumin (BSA) and 0.05% Tween 20 for 30 min under ambient temperature. Next, the blocked membranes were incubated overnight with primary antibodies dissolved in TBS supplemented with 1% BSA and 0.05% Tween-20. After the incubation and washing, the membranes were further incubated with horseradish peroxidase (HRP, 1/1000)-labeled secondary antibodies dissolved in TBS containing 1% BSA and 0.05% Tween-20 for 1 h under ambient temperature. After sufficient washing with TBS supplemented with 0.05% Tween 20, the ECL Western Blotting Detection Reagent (RPN2106, Amersham) was used to develop the protein blots for as long as 30 min. The ImageJ software (US National Institutes of Health) was employed to quantify the band intensity, with b-actin used as the loading reference.

### Statistical analysis

GraphPad Prism 8 was used to analyze the data, which were presented as mean ±standard error of the mean (SEM). Turkey’s multiple comparison test and one-way ANOVA were applied to compare the differences among the different groups. #*P* < 0.05, ## *P* < 0.01 (compared with the NC group) and *P < 0.05, **P < 0.01 (compared with the MC group).

## Results

### Characterization of ASPS-NE

**Figure 1:**
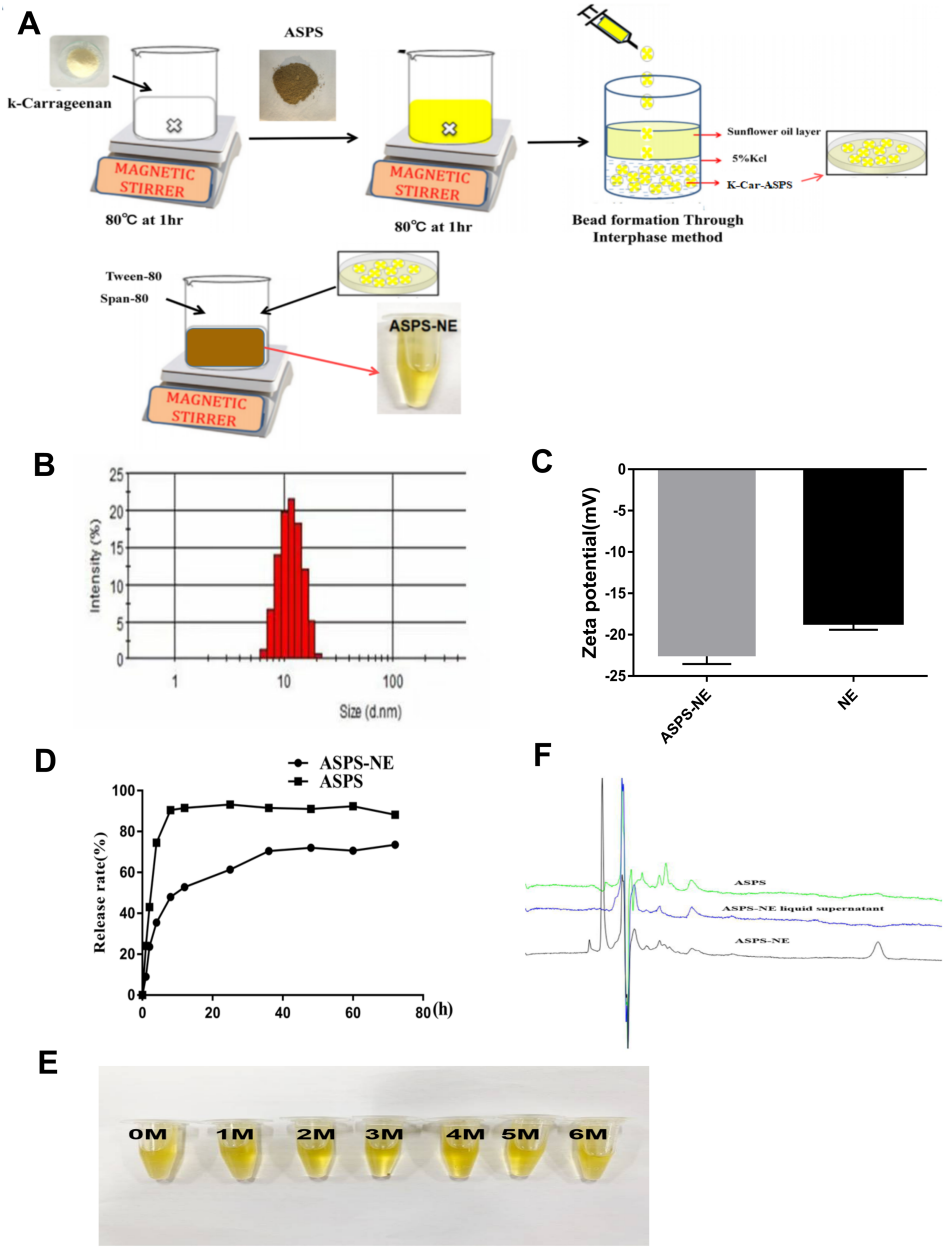
Preparation and characterization of ASPS-NE. (A) The synthetic route of ASPS-NE. (B) The DLS analysis of ASPS-NE in IPM solution. (C) Comparison of the zeta potentials of ASPS-NE and NE. (D) The *in vitro* release curves of ASPS-NE, ASPS. (E) Different time-point photographs of ASPS-NE. (F) HPLC of ASPS, ASPS-NE liquid supernatant, and ASPS-NE.

Excellent physical properties are the prerequisite for the efficacy of ASPS-NE formulations (Carvalho et al., 2020). In the present study, the ASPS-NE formulations were prepared using the Pseudoternary phase diagram method, the production process of ASPS-NE was optimized for PDI, zeta potential, appropriate size, and encapsulation efficiency. The preparation process of the ASPS-NE is illustrated in Fig. 1A. As shown in Figure1B, the ASPS-NE droplets were homogeneously spherical and non-adhesive. The mean particle size and PDI were 35.82 ± 11 nm and 0.195 ± 0.03 (*n* = 4), respectively (Fig. 1B). This result indicated that the size of each nanosphere was distributed in a narrow range. Each ASPS-NE formulation was negatively charged(-23mv) slightly greater than that of the NE (Fig. 1C). The release curve of ASPS-NE was stable *in vitro* compared to that of the ASPS. The release was up to 90% after 60 h, with an obvious sustained-release function (Fig. 1D). After the cold–hot cycle experiment and the light and acceleration experiments, the appearance of ASPS-NE remained clear and transparent, with good fluidity, no floccus or stratification, and no change in the original yellow color (Fig. 1E). As depicted in Fig. 1F, ASPS-NE exhibited standard characteristic peaks similar to those of ASPS.

### Effects of ASPS-NE on Splenocyte proliferation *in vitro*

**Figure 2:**
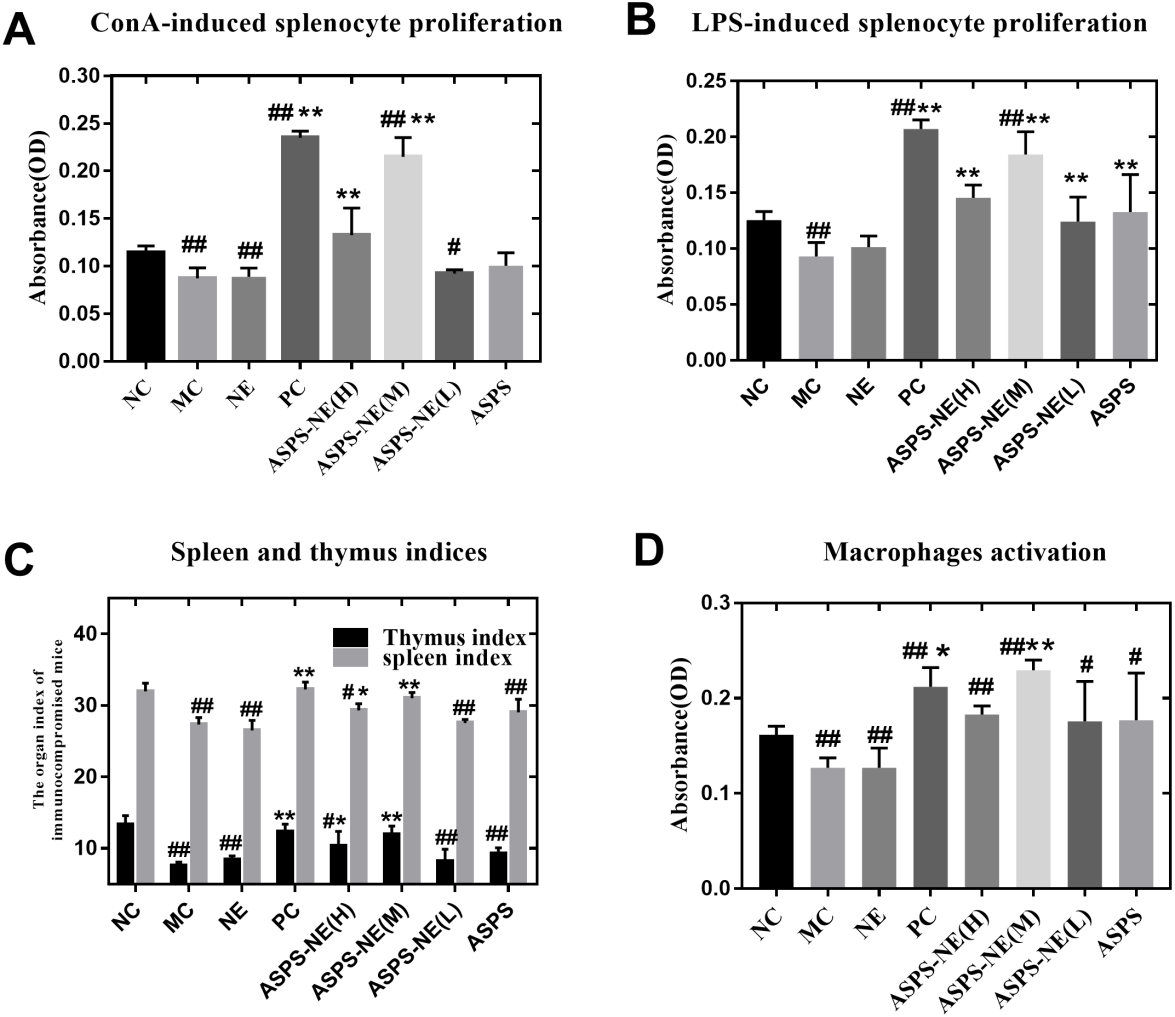
Results of Splenocyte proliferation, organ index, and macrophage phagocytosis in the immunocompromised mice. (A) Effect of ASPS-NE on ConA-induced mice splenocyte proliferation. (B) Effect of ASPS-NE on LPS-induced mice splenocyte proliferation. (C) Spleen and thymus ind ices of the immunosuppressive mice. (D) Effect of ASPS-NE on the macrophages activation in the immunosuppressive mice. # *P* < 0.05, ## *P* < 0.01 (compared with the NC group) and * *P* < 0.05, ** *P* < 0.01 (compared with the MC group).

ASPS has been previously demonstrated to improve lymphocyte proliferation and exhibit potent immunomodulatory activity *in vitro* as well as *in vivo* (Han et al., 2014). The spleen plays a vital role in the immune reactions occurring in the body. In addition, the spleen cells contain a large number of lymphocytes and macrophages (Steiniger et al., 1997). In order to evaluate the immunomodulatory effect of the prepared ASPS-NE formulations, their effects on splenocyte proliferation were evaluated *in vivo* as described in a previous study (Luo et al., 2020). As depicted in Figs. 2A and 2B, the lymphocytes were treated with the mitogens ConA (10 µg/ml final concentration) (Fig. 2A) and LPS (5 µg/ml final concentration) (Fig. 2B) *in vitro*. According to the results, the lymphocyte proliferation was observed to have decreased markedly in the MC, NE group compared to the normal control group (*P* < 0.01). In addition, the combination of ConA and LPS promoted the splenocyte proliferation in the ASPS-NE group, indicating that ASPS-NE could promote the proliferation of splenocytes.

### ASPS-NE promote the organ index and macrophages activation

On the 7th day, the spleen and thymus samples from each group were dissected and weighed accurately. As depicted in Fig. 2C, the indices of spleen and thymus in the MC, NE group were markedly decreased relative to the NC group. On the contrary, the indices of spleen and thymus in the ASPS-NE treatment group were remarkably increased relatively, especially in the ASPS-NE(M)group. Therefore, the neutral red uptake experiment was conducted next to examine the effect of ASPS-NE on macrophages activation to estimate the immunomodulation effect of ASPS-NE. As depicted in Fig. 2D, macrophages activation was significantly decreased in the MC group compared to the NC group (*P* < 0.01), while the macrophage activations in the ASPS-NE (M and H) and PC groups was remarkably increased relative to the MC group (*P* < 0.01). Therefore, it was inferred that ASPS-NE improved the activation of peritoneal macrophages in the immunosuppressive mice model. By researched the immunological enhancement effect of ASPS-NE formulations in immunosuppressive mices, NE groups was no significant difference relative to the MC group. Therefore, the immunoregulatory effect of ASPS-NE in the immunosuppressed mice was possibly closely associated with macrophage activation.

### ASPS-NE enhance the proliferation of peripheral blood T lymphocyte subsets of CD4^+^/CD8^+^ T cells in immunosuppressive animal model

**Figure 3:**
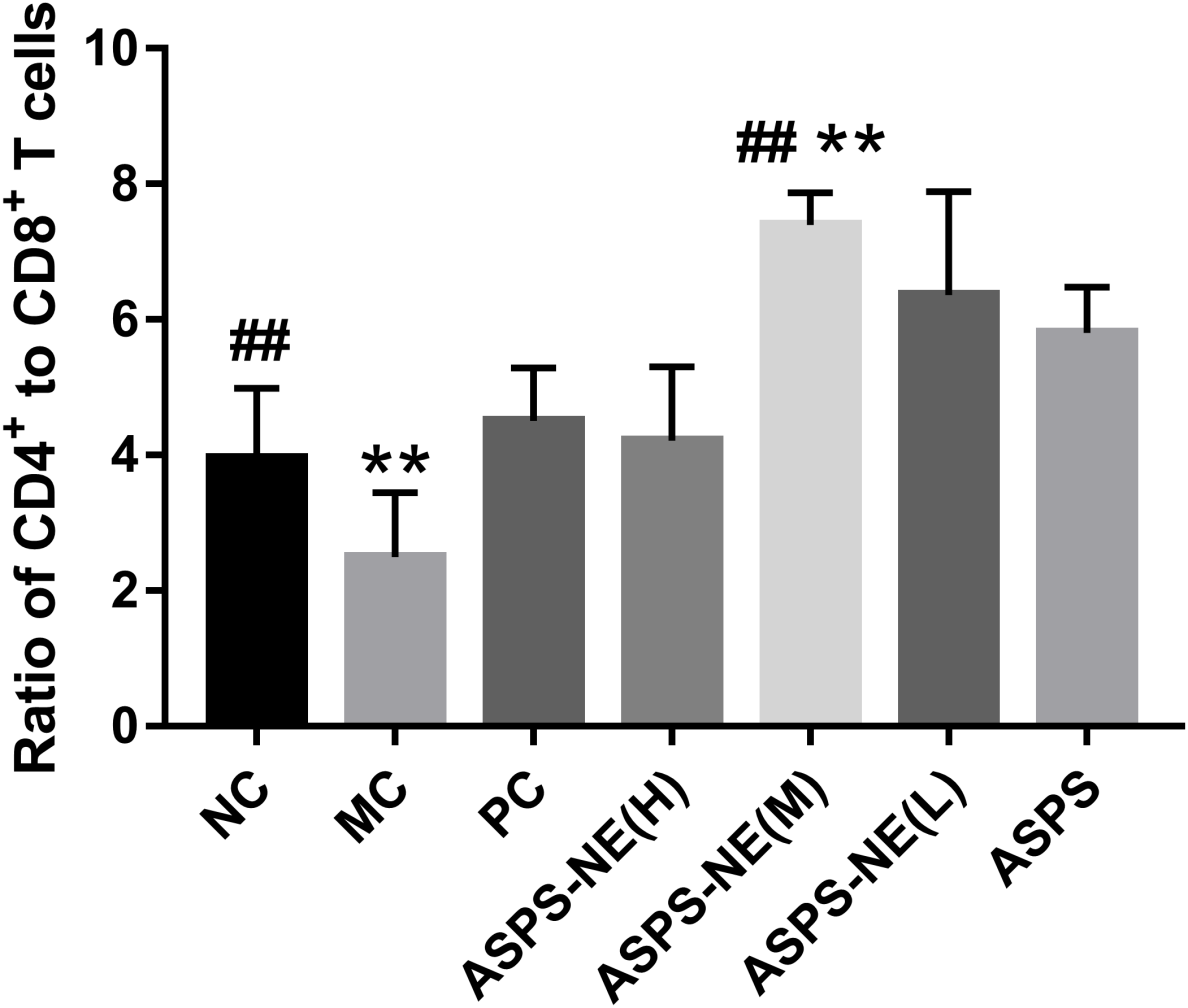
The ratio of CD4^+^ to CD8^+^ T cells in NC, MC, PC, ASPS NE(H), ASPS NE(M), ASPS NE(L), ASPS. # *P* < 0.05, ## *P* < 0.01 (compared with the NC group) and * *P* < 0.05, ** *P* < 0.01 (compared with the MC group).

The activation of antigen-specific CD4^+^ and CD8^+^ cytotoxic T lymphocytes (CTLs) is considered a marker of cellular immunity. Therefore, flow cytometry analysis was performed to detect the CD4^+^ and CD8^+^ T cell ratios in the peripheral blood samples from immunosuppressed mice. As visible in Fig. 3, the MC group presented a lower percentage of CD4^+^/CD8^+^ T cells compared to the NC group (*P* < 0.01), and ASPS-NE induced great proportions of CD4^+^/CD8^+^T cells relative to the MC group (*P* < 0.01). These results were consistent with the findings of the peripheral blood proliferation analysis. Therefore, it was inferred that mice under ASPS-NE formulation stimulation exhibited induced potent cellular immunity, while those stimulated with Cy, ASPS, and the other control formulations did not.

### ASPS-NE promote cell release of cytokines through immune enhancement effect

**Figure 4:**
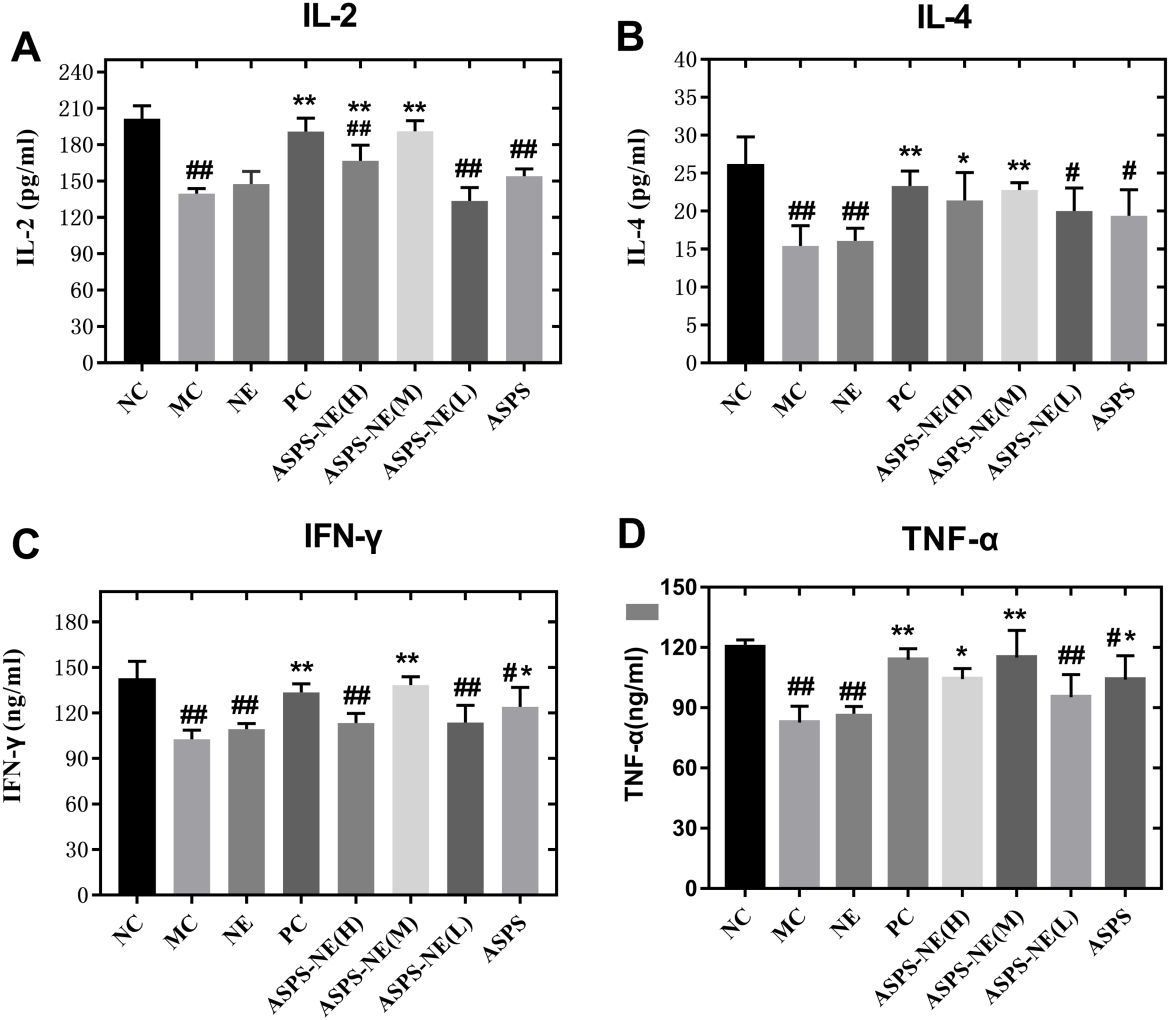
The change s in the serum cytokine levels in each group (A) IL-2, (B) IL-4, (C) IFN-γ, and (D) TNF-α. # *P* < 0.05, ## *P* < 0.01 (compared with the NC group) and * *P* < 0.05, ** *P* < 0.01 (compared with the MC group).

In comparison to the other dosage formulations, the ASPS-NE mainly produced Th1/Th2 antibody reactions (dominated by Th1) in mice serum. Therefore, we further studied whether the ASPS-NE formulations induced cytokine transformation with Th1 dominance. The serum Th1 cytokines (IFN-γ, TNF-α, and IL-2) and Th2 cytokines (IL-4) were collected and analyzed through ELISA. As visible in Figs. 4A–4D, the MC group generated much lower Th1 cytokine contents compared to the NC, NE group (*P* < 0.01). The ASPS-NE (M) group exhibited a markedly increased production of Th1 cytokine contents relative to the MC group (*P* < 0.01). However, the Th2 cytokine content produced in the ASPS-NE group was markedly increased relative to the MC group (*P* < 0.01). NE groups was no significant difference relative to the MC group. The Th1 cytokine contents demonstrated similar trends. Taken together, ASPS-NE exhibited the production of higher Th1 and Th2 cytokine contents compared to the other formulations, suggesting that NE elicits a stronger immune response.

### ASPS-NE enhanced immune effects *via* P65/JNK/ikk α activation in peritoneal macrophages

**Figure 5:**
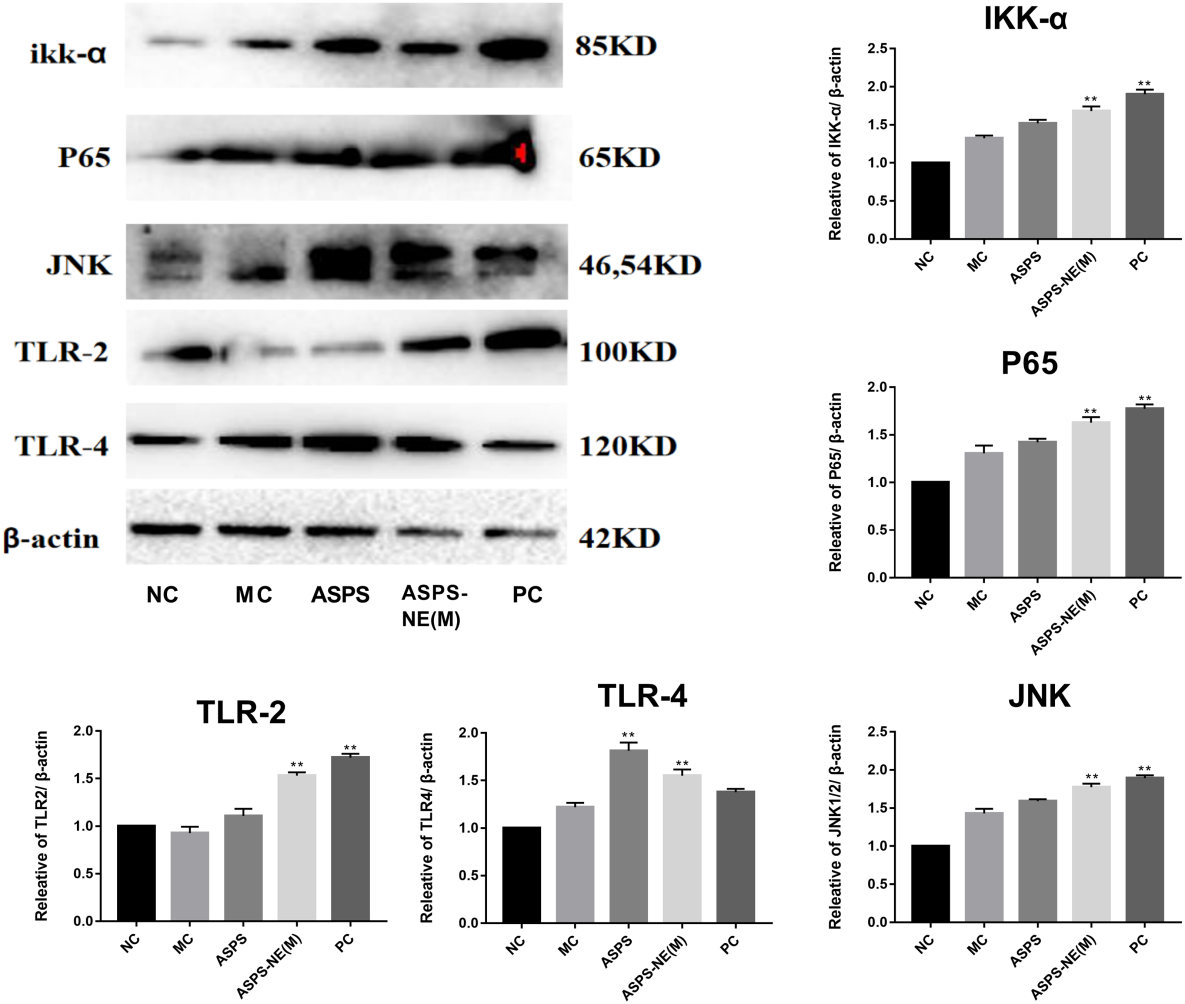
Effects of ASPS-NE on ikk α, P65, JNK, TLR-2,TLR-4 protein levels in peritoneal macrophages. Expression normalized to β-actin detected in Western blotting. # *P* < 0.05, ## *P* < 0.01 (compared with the NC group) and * *P* < 0.05, ** *P* < 0.01 (compared with the MC group).

Previous studies have reported the immune enhancement effects of ASPS-NE *in vitro*. These effects of ASPS-NEs were further demonstrated in the present study using peritoneal macrophages. The results of the Western blot assays, presented in Fig. 5, revealed that ASPS-NE(M) could activate the expression of P65/JNK/ikk α in a dose-dependent compared to the other groups (*P* < 0.01 or *P* < 0.05).

### ASPS-NE enhanced immune effects *via* regulation of Th1/Th2 ratio in peritoneal macrophages

**Figure 6:**
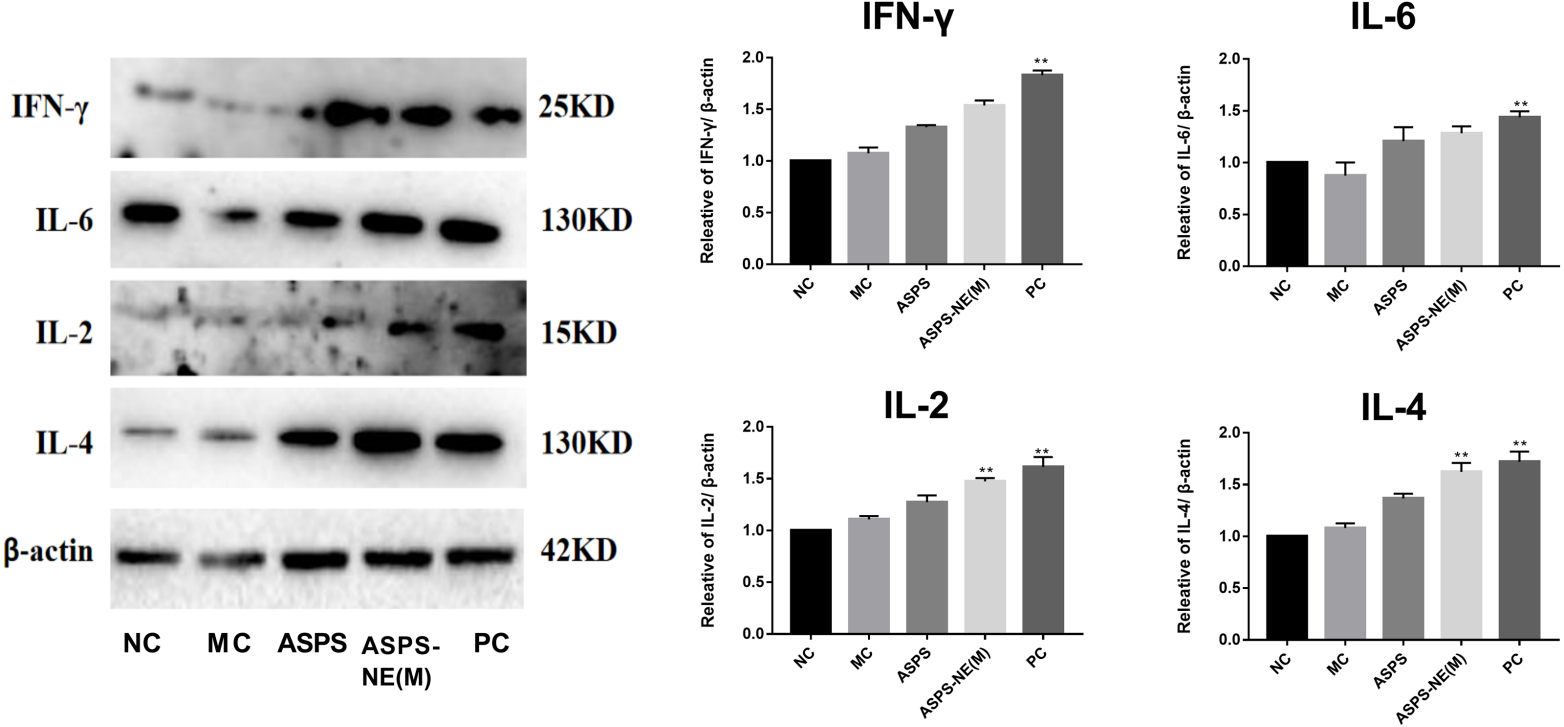
Effects of ASPS-NE on IL-2, IL-4, IL-6 , and IFN- γ protein levels in peritoneal macrophages. Expression normalized to β-actin detected in Western blotting . # *P* < 0.05, ## *P* < 0.01 (compared with the NC group) and * *P* < 0.05, ** *P* < 0.01 (compared with the MC group).

As evidenced by the Western blot assay results presented in Fig. 6, the ASPS-NE(M) induced higher IFN- γ and IL-2/-4/-6 levels compared to the other groups (*P* < 0.01 or *P* < 0.05). According to this finding, ASPS-NE enhanced the immune effects through the regulation of the Th1/Th2 cytokines in the peritoneal macrophages.

## Discussion

In the present study, ASPS-NE formulations were prepared and characterized by determining their physicochemical characteristics, such as stability, structure, shape, and particle size. The zeta potential and TEM analyses revealed that all the formulations had narrow zeta potential polarity and size distribution. The prepared ASPS-NE exhibited the particle size, PDI, and zeta potential of 35.82 ± 11 nm, 0.195 ± 0.03, and −23 mV, respectively, which were sufficient for the mutual interaction of the nanospheres, thereby rendering sustained stability and preventing particle aggregation.

In addition, the splenocyte-induced cell-mediated immunity in the inoculated mice was evaluated, and the cytokine contents were determined to explore whether the nanospheres were able to induce an inflammatory cell-mediated immune reaction. It was observed that, in mice, the Th1 cytokines (including IL-2, IFN- γ, and TNF-α) induced the CTL response and promoted the proliferation of activated B cells *via* surface receptors, in addition, to directly inducing IgG2a production. Moreover, IL-4, a major Th2 cytokine, was observed to enhance the viability and proliferation of B cells (Niu et al., 2015; Villalva et al., 2020). Furthermore, both Th1 and Th2 cytokines exhibited higher secretion in comparison to that in the other groups (Ye et al., 2017).

The proliferation of lymphocytes and splenocytes is an important hallmark of mitogen- and antigen-induced activation of the humoral and cellular immune responses, both of which represent the classic non-specific immune response that has been investigated extensively in previous studies (Ferraris et al., 2019). In the present study, the proliferation of splenocytes was investigated using the CCK-8 assay, which revealed that with the increasing of the concentration of polysaccharide , the proliferation of polysaccharide nanoemulsion was also enhanced, showing a certain dose-dependent. ASPS-NE (M) had the highest contribution in promoting splenocyte proliferation. asps-ne could effectively promote the proliferation of splenic lymphocytes in mice, and can synergistically stimulate B lymphocytes and T lymphocytes with LPS and ConA. In particular, the ASPS-NE (M) group had the best proliferation effect. By researched the immunological enhancement effect of ASPS-NE formulations in immunosuppressive mice , NE groups was no significant difference relative to the MC group. Similarly,there has been reported that polysaccharides from black ganoderma lucidum can jointly promote the proliferation of mice lymphocytes with ConA and LPS, and show a certain dose effect (Zhang et al., 2014).

CD4^+^ T cells are the key indicators of cell-mediated immunity as they regulate numerous immune responses, such as offering assistance to the additional immunocytes and secreting the necessary cytokines to resist infections (O’Garra, Gabryšová & HJNi, 2011; Swain, McKinstry & Strutt, 2012). The cytotoxic CD8^+^ T cells execute their functions through their antigen presentation property. The CD4 / CD8 ratio is an immunomodulatory indicator.

In order to study the ability of the ASPS-NE to activate antigen-specific CD3^+^ CD4^+^/CD3^+^ CD8^+^ T cells, this ability was compared among the different groups. As presented in Fig. 3, compared with the nc group, the ratio of cd4+/cd8+ in the model group was significantly decreased (*P* < 0.01), indicating that the model was successfully constructed. compared with the model group, the ratio of CD4+/CD8+ In the ASPS-NE (M), ASPS-NE (L) and pc group was significantly increased (*P* < 0.01). The results indicated that ASPS-NE (M), ASPS-NE (L) and pc groups significantly improved the immune function of immunosuppressed mice, especially the best effect was ASPS-NE (M). indicating that T cell proliferation might modulate the activities of CTLs and B cells following ASPS-NE immunization.

Macrophages, which are derived from monocytes, perform vital functions in the innate and adaptive immune responses (Li et al., 2020; Yao et al., 2018). Macrophage phagocytosis represents a vital initial link in an immune reaction. The activity of macrophages is evaluated in terms of their increased phagocytosis (Bi et al., 2018; Sheng et al., 2017). The results of the present study revealed that ASPS-NE could enhance the phagocytosis of peritoneal macrophages in the immunosuppressive mice models, with the ASPS-NE (M) group exhibiting a stronger effect compared to the MC group. Studies have shown that the nanoemulsion itself has no immune-enhancing effect.Therefore, it was inferred that ASPS-NE could activate adaptive as well as innate immunity through the activation of macrophages.

Cytokines are low-molecular-weight proteins, which could originate from the immunocytes, such as macrophages, T cells, and B cells, and exert different effects on adaptive, innate, and self immunity (Dehghan et al., 2018). Moreover, cytokines perform crucial functions in pathogen resistance. Among all the cytokines, IFN- γ , IL-2/6, and TNF- α play the most important roles in resisting the intracellular pathogens (Cao et al., 2016; Zheng et al., 2018). According to the results obtained in the present study, the ASPS-NE and PC groups promoted the mRNA levels of TNF- α, IL-2/4, and IFN- γ compared to the MC group (*P* < 0.01), with ASPS-NE exhibiting the strongest stimulation. According to these findings, it was inferred that ASPS-NE could restore the reduced expression levels of cytokines by stimulating macrophages. ASPS-NE could also activate the expression of P65, JNK, and ikk α, along with regulating the Th1/Th2 cytokines. These findings demonstrated that ASPS-NE had the potential for application in drug delivery and induction of potent and sustained immune reactions.

## Conclusion

In summary, the present study reported the preparation of ASPS-NE formulation, which is a natural immune modulator with an average molecular weight of 5 × 10^4^ Da. According to the results obtained in the present study, ASPS-NE exhibit a strong immunomodulatory effect and reverse immunosuppression by promoting the proliferation of various immunocytes and the phagocytosis of peritoneal macrophages. Moreover, ASPS-NE induced several cytokines (IL-2, IL-6, TNF-α, and IFN-γ) involved in the intrinsic peritoneal macrophages-dependent TLR4-NF-κB signal transduction pathway-induced immunoregulation signaling. In addition, ASPS-NE partially activated the splenocytes while relieving spleen damage. The results may vary according to the animal species. the findings of this study are likely to generalise to other species.

Overall, the results obtained in the present work indicated that ASPS-NE have the potential to serve as a biological additive and an efficient approach to improve immunity in the immunocompromised cases in animal husbandry.

## Supplemental Information

10.7717/peerj.12575/supp-1 Supplemental Information 1 Full ARRIVE 2.0 checklist

10.7717/peerj.12575/supp-2 Supplemental Information 2 Results of Splenocyte proliferation, organ index, and macrophage phagocytosis in the immunocompromised mice (A) Effect of ASPS-NE on ConA-induced mice splenocyte proliferation. (B) Effect of ASPS-NE on LPS-induced mice splenocyte proliferation. (C) Spleen and thymus indices of the immunosuppressive mice. (D) Effect of ASPS-NE on the phagocytosis of peritoneal macrophages in the immunosuppressive mice. # *P* < 0.05, ## *P* < 0.01 (compared with the NC group) and * *P* < 0.05, ** *P* < 0.01 (compared with the MC group).

10.7717/peerj.12575/supp-3 Supplemental Information 3 Surface expression of CD4+/CD8+ T cells in NC, MC, PC, ASPS-NE(H), ASPS-NE(M), ASPS-NE(L) and ASPS, as determined by flow cytometry

10.7717/peerj.12575/supp-4 Supplemental Information 4 The changes in the serum cytokine levels in each group (A) IL-2, (B) IL-4, (C)IFN-γ, and (D) TNF-α # *P* < 0.05, ## *P* < 0.01 (compared with the NC group) and * *P* < 0.05, ** *P* < 0.01 (compared with the MC group).

10.7717/peerj.12575/supp-5 Supplemental Information 5 Numerical data in Figs. 1C, 1D, 1E, 1G; Figs. 2A–2D, 3, 4A–4D; plots in Figs. 5 & 6

10.7717/peerj.12575/supp-6 Supplemental Information 6 Figs. 5 and 6 full-length uncropped blots

## Additional Information and Declarations

### Data Availability

The following information was supplied regarding data availability: The raw measurements and western blots are available in the Supplemental Files.
