# Supplementary figures and images for: Macrophage immunomodulatory activity of Acanthopanax senticousus polysaccharide nanoemulsion via activation of P65/JNK/ikkαsignaling pathway and regulation of Th1/Th2 Cytokines
Source: PeerJ. 2021 Dec 24;9:e12575. doi: 10.7717/peerj.12575 (PMC8711278; doi:10.7717/peerj.12575)

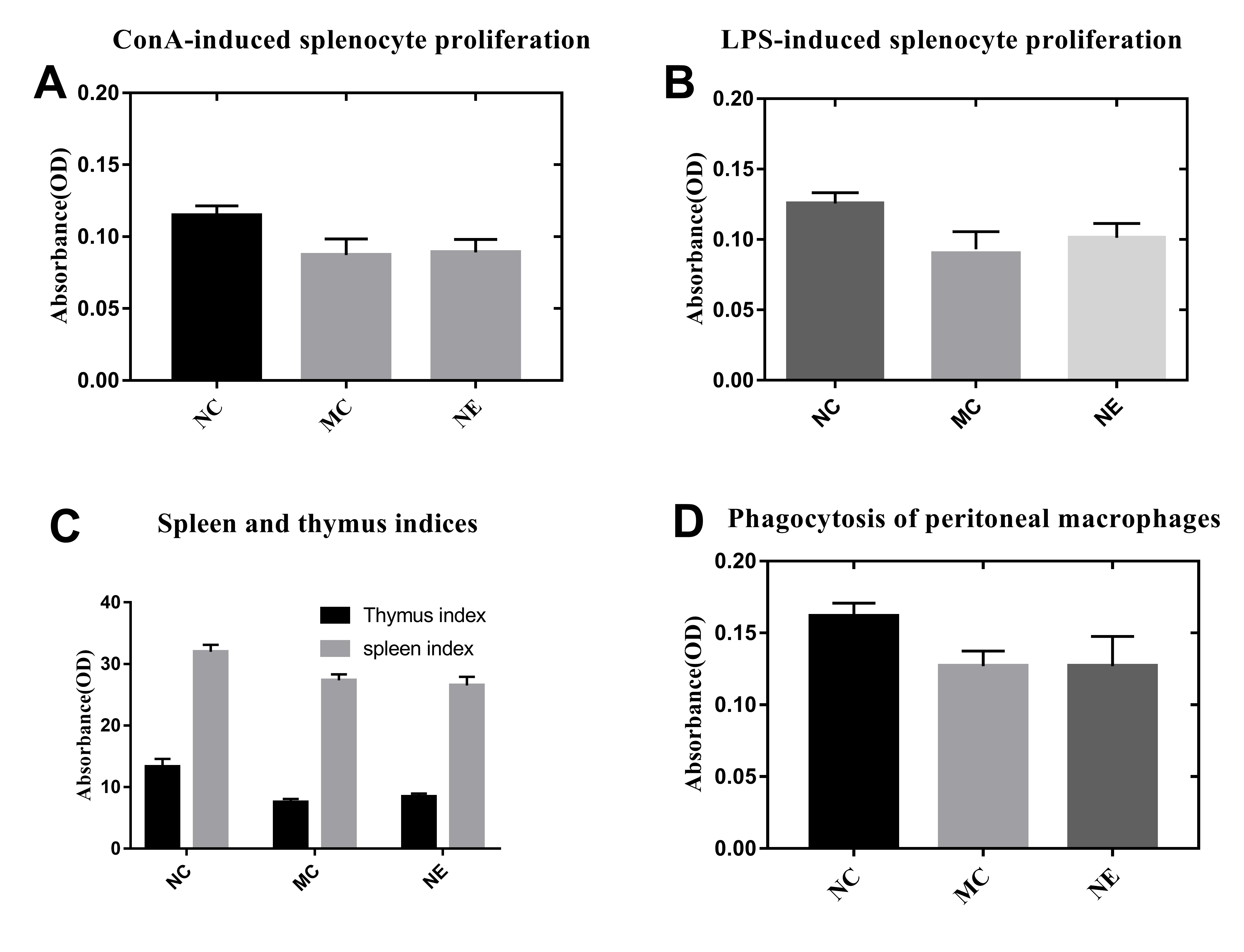

Supplement: Supplemental Information 2 — (A) Effect of ASPS-NE on ConA-induced mice splenocyte proliferation. (B) Effect of ASPS-NE on LPS-induced mice splenocyte proliferation. (C) Spleen and thymus indices of the immunosuppressive mice. (D) Effect of ASPS-NE on the phagocytosis of peritoneal macrophages in the immunosuppressive mice. # P < 0.05, ## P < 0.01 (compared with the NC group) and * P < 0.05, ** P < 0.01 (compared with the MC group). [file peerj-09-12575-s002.png]

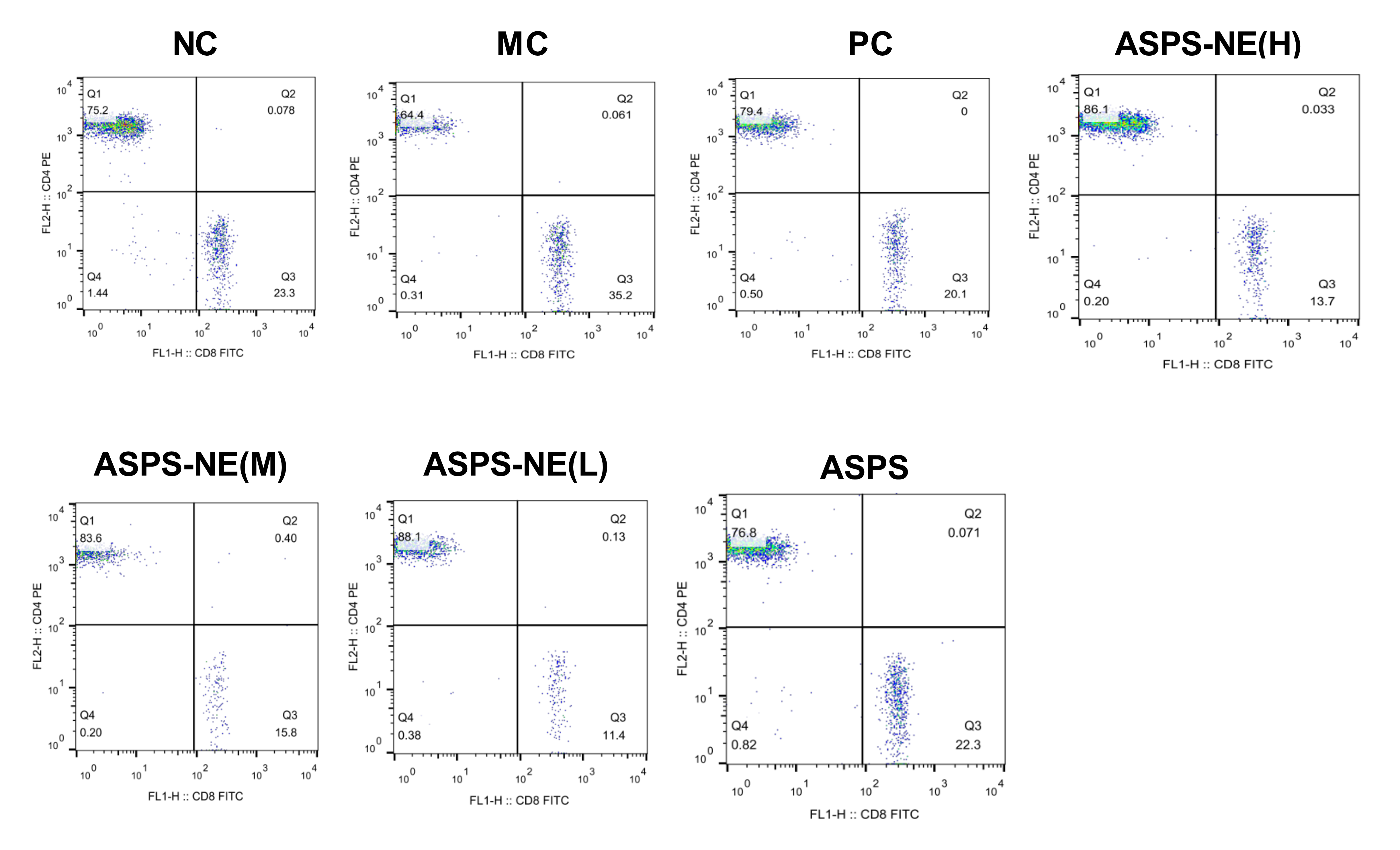

Supplement: Supplemental Information 3 [file peerj-09-12575-s003.png]

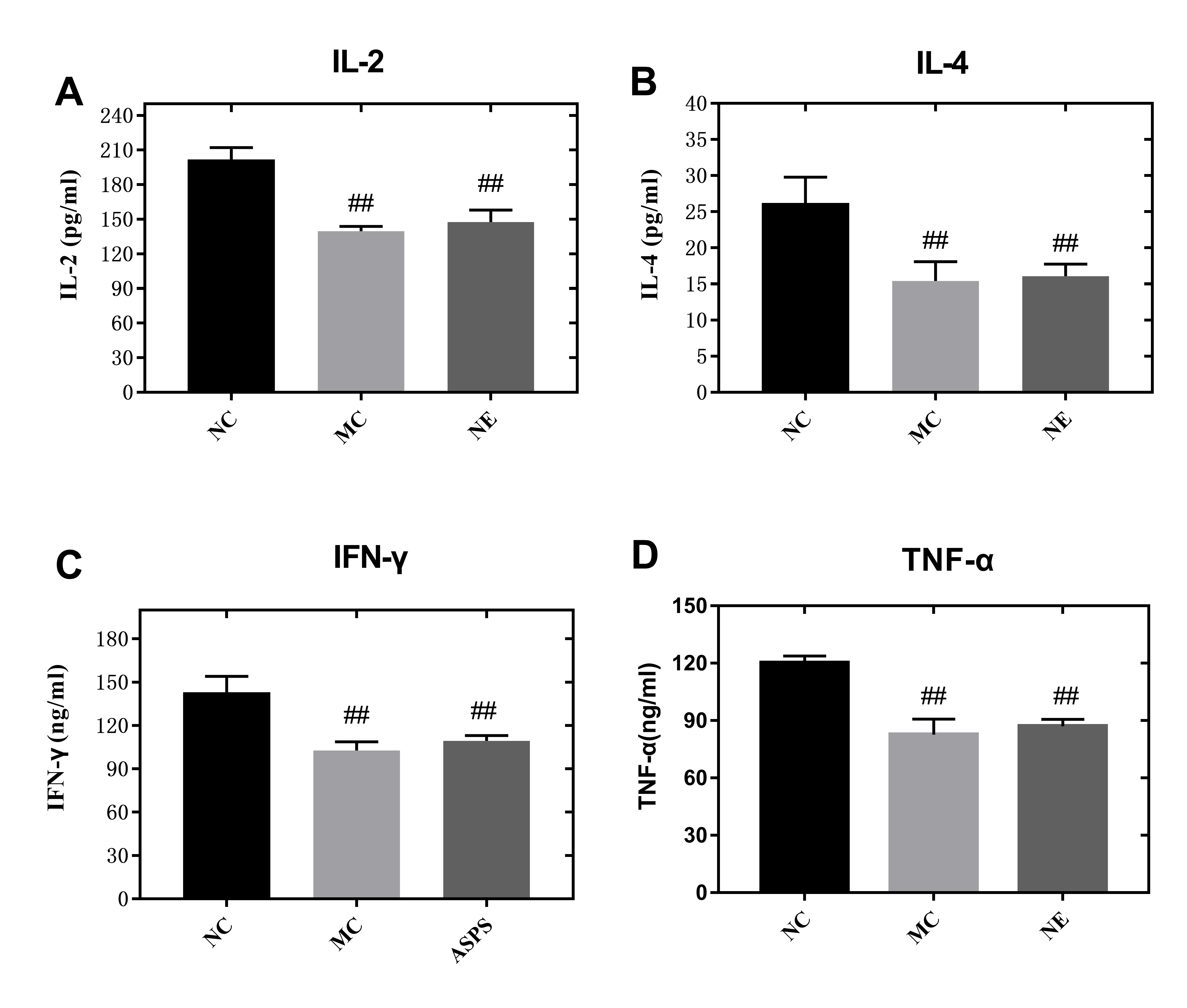

Supplement: Supplemental Information 4 [file peerj-09-12575-s004.png]
